# Leisure-Time Physical Activity Has a More Favourable Impact on Carotid Artery Stiffness Than Vigorous Physical Activity in Hypertensive Human Beings

**DOI:** 10.3390/jcm11185303

**Published:** 2022-09-08

**Authors:** Olga Vriz, Lucio Mos, Paolo Palatini

**Affiliations:** 1 Cardiac Centre, King Faisal Specialist Hospital and Research Center, Riyadh 11211, Saudi Arabia; 2 School of Medicine, Alfaisal University, Riyadh 11533, Saudi Arabia; 3 Department of Cardiology, San Antonio Hospital, 33038 San Daniele del Friuli, Italy; 4 Department of Medicine, University of Padova, 35128 Padua, Italy

**Keywords:** exercise, physical activity, carotid stiffness, pulse wave velocity, hypertension

## Abstract

**Aim.** To assess the effect of leisure time versus vigorous long-term dynamic physical activity (PA) on carotid stiffness in normotensive versus hypertensive subjects. **Methods.** The study was conducted on 120 leisure-time exercisers and 120 competitive athletes. One hundred and twenty sedentary subjects served as controls. In addition, participants were classified according to whether their systolic blood pressure was ≥130 mmHg (hypertensives, *n* = 120) or normal (normotensives, *n* = 240) according to the ACC/AHA 2017 definition. Carotid artery stiffness was assessed with an echo-tracking ultrasound system, using the pressure-strain elastic modulus (EP) and one-point pulse wave velocity (PWVβ) as parameters of stiffness. **Results.** The effect of the two levels of PA differed in the normotensives and the hypertensives. Among the normotensives, there was an ongoing, graded reduction in EP and PWVβ from the sedentary subjects to the athletes. By contrast, among the hypertensives, the lowest levels of EP and PWVβ were found among the leisure-time PA participants. EP and PWVβ did not differ between the hypertensive sedentary subjects and the athletes. A significant interaction was found between PA and BP status on EP (*p* = 0.03) and a borderline interaction on PWVβ (*p* = 0.06). In multiple regression analyses, PA was a negative predictor of EP (*p* = 0.001) and PWVβ (*p* = 0.0001). The strength of the association was weakened after the inclusion of heart rate in the models (*p* = 0.04 and 0.007, respectively). **Conclusions.** These data indicate that in people with hypertension, leisure-time PA has beneficial effects on carotid artery stiffness, whereas high-intensity chronic PA provides no benefit to vascular functions.

## 1. Introduction

A large number of studies have shown that regular aerobic physical activity (PA) is beneficial for both the prevention and treatment of hypertension [1], diabetes [2], and ischemic heart disease [3]. Thus, most international guidelines recommend that people engage in at least 150 to 300 min/wk of moderate PA, 75 to 150 min/wk of vigorous PA or a balanced combination of the two [1,2,3]. The protective role of regular PA in cardiovascular diseases has been attributed to several different factors, including a decrease in blood pressure (BP), heart rate, and body mass index (BMI), an improvement in the lipid profile and an increase in insulin sensitivity [4,5,6]. These mechanisms also seem to be responsible for the beneficial effects of PA on arterial distensibility [7,8]. Indeed, several cross-sectional and longitudinal studies have demonstrated that regular PA is associated with a decrease in large artery stiffness. In a meta-analysis including 42 clinical trials (*n* = 1627 participants), Ashor et al. [9] showed that aerobic exercise caused a significant reduction in pulse wave velocity and augmentation index, an effect that was larger in participants with stiffer arteries. However, there are concerns about the potentially harmful effects of an excessive amount of high-intensity PA on the cardiovascular system [10]. In particular, whether engaging in vigorous competitive aerobic sports provides a greater benefit than leisure-time PA on cardiovascular health or whether it may be even more harmful is not known. It might well be that a high level of chronic PA causes a detrimental effect on vascular structure and function in the long run in people with hypertension. Thus, the aim of this study was to assess the effect of leisure-time, long-term aerobic PA versus vigorous PA on carotid artery stiffness in relation to blood pressure status. A secondary objective was to evaluate whether, and to what extent, the effect of PA on carotid stiffness was mediated by the PA-induced heart rate reduction.

## 2. Methods

### 2.1. Study Population

The present study was a cross-sectional analysis of data collected from 2009 to 2017 [11]. Subjects were selected from among those referred to the outpatient clinic of the Cardiology Department of the San Antonio Hospital, San Daniele del Friuli, Udine (Italy), for work eligibility or competitive athletics participation and from healthy subjects who wanted a cardiologic work-up. Apparently healthy subjects without important cardiovascular risk factors or overt cardiovascular disease, taking no medications, were also included. Among these, 120 consecutive athletes engaging in aerobic competitive sports and 120 subjects of similar age who performed leisure-time aerobic PA for at least 2 years were considered. One hundred and twenty sedentary subjects were used as controls. To define the PA category, we used previously published criteria [4] using a standardized questionnaire. Briefly, activities were classified on the basis of relative intensity. Individuals were categorized as sedentary (class 0) if they did not regularly perform any sports activity; leisure-time exercisers (class 1) if they performed leisure physical activities such as running, jogging, cycling, swimming, soccer, tennis and so on at least once a week without participating in competitions; and athletes (class 2) if they performed aerobic sports and participated in competitions requiring an eligibility check-up. The exclusion criteria covered all cardiovascular diseases, diabetes, chronic kidney disease, other serious diseases, hypertension on antihypertensive treatment, and any other condition requiring chronic medication. All participants underwent a standard initial evaluation, including a thorough medical history inquiry and careful physical examination, using the same procedures for all [11,12]. Data on medical history, dietary habits, and cigarette smoking (yes or no) were collected from all participants. All methods were performed in accordance with the relevant guidelines and regulations according to the Declaration of Helsinki. Ethical approval was obtained from the local Ethics Committee, and informed consent was provided by each participant.

### 2.2. Procedures

Office brachial BP and heart rates were measured 6 times over two visits using a validated oscillometric semiautomatic sphygmomanometer on the dominant arm, in the supine position, and a quiet room [4]. On the basis of the mean of the 6 BP readings, participants were classified according to whether their systolic BP was ≥130 mmHg (hypertensives, *n* = 120) or normal (*n* = 240) according to the definition of hypertension by 2017 ACC/AHA guidelines [13]. The procedures used to assess local carotid stiffness were previously published [11,12]. Briefly, measurements were made at the level of the left common carotid artery, at one point between 1 and 2 cm, before its bifurcation using a high-definition echo-tracking ultrasound system (Alpha 10, Aloka, Tokyo, Japan) with a multifrequency 5–13 MHz linear probe, which uses raw radiofrequency signals that are based on video signals with an accuracy of 0.01 mm. The optimal angle between the ultrasound beam and the vessel wall for diameter change measurements by echo-tracking is 90°, but as the blood-flow velocity is perpendicular to the beam, it cannot be detected. Thus, a different independent steerable ultrasound beam was used. Both beams were steered to intersect at the center of the range gate. The ultrasound beam steering angle can be changed from −30° to +30° with 5° angular increments. The echo-tracking gates were manually set at the boundaries between the intima and media of the anterior and posterior walls. The rate gate for velocity measurements was automatically positioned at the center of the diameter using echo-tracking gates. Flow velocity was corrected for the angle between the ultrasound beam and the blood flow velocity vector. Brachial cuff pressure was measured just before starting ultrasound imaging and was entered into the system for the calculation of carotid stiffness indices [14]. At least five consecutive beats were averaged to obtain a representative waveform. The two parameters of stiffness taken into account were the pressure-strain elastic modulus (EP) and one-point pulse wave velocity (PWVβ). The formulas used for the calculation of the carotid arterial stiffness parameters were:

EP = (Ps − Pd)/[(Ds − Dd)/Dd] and PVWβ = ln(Ps/Pd)/[(Ds − Dd)/Dd]

where Ds and Dd refer to carotid arterial systolic and diastolic diameter; Ps and Pd refer to systolic and diastolic brachial BP (used as a surrogate of carotid systolic and diastolic BP). Inter and intra variability and repeatability of carotid stiffness parameters were previously published [15].

### 2.3. Statistics

All continuous data were normally distributed according to the Shapiro–Wilk normality test and were presented as means (±SD). Comparisons between the three groups were carried out using a two-way analysis of covariance, using the BP status and PA category as fixed factors and age, sex, BMI, diastolic BP and smoking as covariates. Variables with a *p* < 0.15 in univariate analysis were entered into a multivariate linear regression, which was used to investigate the association of carotid stiffness with PA (yes or no) and heart rate, thereby controlling for confounders. To compare regression model fit, we used the Akaike Information Criteria (AIC, −2Log-likelihood +2k, where k is the number of parameters estimated) and Schwarz’s Bayesian Information Criterion (BIC, −2∗Log-likelihood + k∗log (n)) [16,17]. For both AIC and BIC criteria, the model providing the smallest values is considered to have the best fit [16,17]. In addition, a measure that can be used to compare survival models is the delta AIC (Δi). This is a measure of each model’s AIC relative to the model with the smallest AIC. As a rule for interpretation, a Δi < 2 suggests substantial evidence for the model, as values between 3 and 7 indicate that the model has considerably less support, whereas a Δi > 10 indicates that the model is very unlikely [18]. Categorical data were compared with the chi^2^ test or Fisher’s exact test and were presented as a percentage. Analyses were performed using a significance level of α = 0.05 (2-sided).

## 3. Results

The main characteristics of the study participants are shown in Table 1. Compared with sedentary subjects, athletes were younger (*p* = 0.03). No age difference was found between the two active groups (*p* = 0.36). No differences in sex distribution, systolic BP and diastolic BP were found between the three groups. The prevalence of smokers was lower among the athletes than the leisure-time PA group. BMI and heart rate progressively decreased from the sedentary subjects to the athletes (Table 1).

### 3.1. Carotid Stiffness Parameters

**Figure 1:**
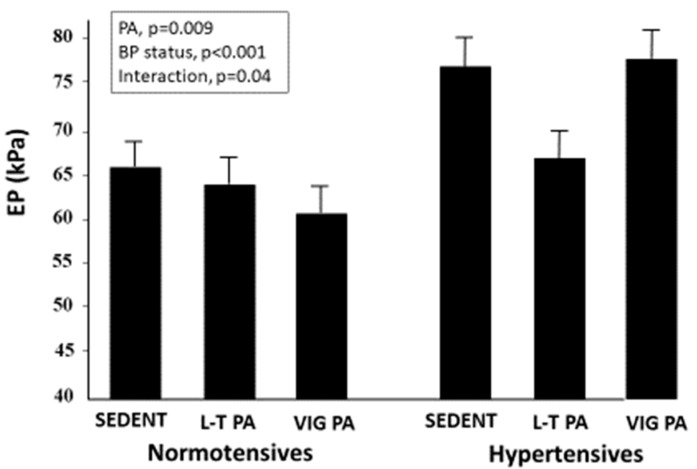
Pressure-strain elastic modulus (EP) in the study participants stratified according to physical activity level and blood pressure status. Sedent indicates sedentary subjects; L-T PA indicates leisure-time physical activity; VIG PA indicates vigorous physical activity (competitive athletes).

EP was lower in both active groups than in the sedentary subjects (*p* < 0.03) and in the normotensives than the hypertensives (*p* < 0.001). However, in a two-way ANCOVA adjusted for age, sex, BMI, diastolic blood pressure and smoking, a different pattern was observed among the normotensives and the hypertensives (Figure 1). In the former, a graded decrease in EP was observed with increasing PA levels, whereas, in the latter, the lowest EP values were observed in the leisure-time activity group. A significant effect on EP was found for both blood pressure status (*p* < 0.001) and PA (*p* = 0.009) with a significant interactive effect of the two factors (*p* = 0.04).

**Figure 2:**
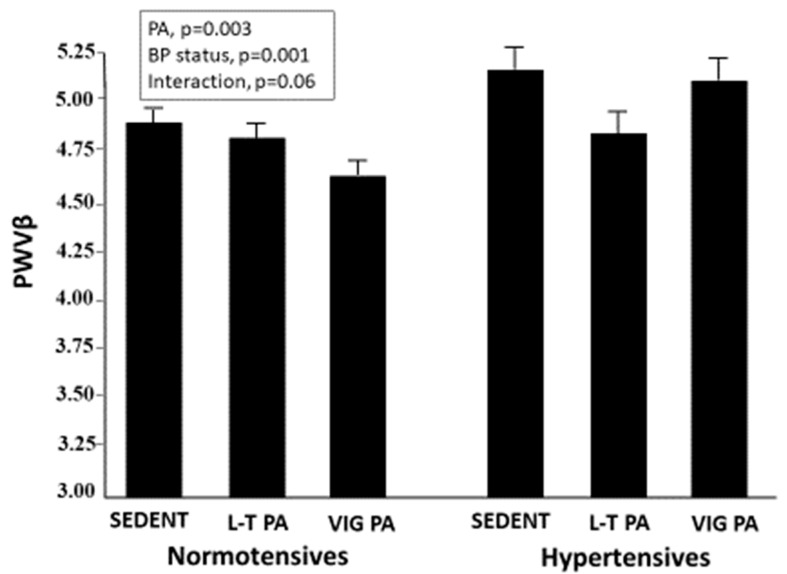
One-point pulse wave velocity in the study participants stratified according to physical activity level and blood pressure status. Legends as in Figure 1.

Similar results were found for PWVβ. PWVβ was lower in both athletes and leisure-time PA participants than in sedentary subjects (*p* < 0.02) and was lower in the normotensives than the hypertensives (*p* < 0.001). Again, in a two-way ANCOVA, a linear relationship was found in the normotensive group, and a U-shaped relationship was found in the hypertensive participants (Figure 2). A significant effect was found for both blood pressure status (*p* = 0.001) and PA (*p* = 0.003) on PWVβ, with a borderline interactive effect of the two factors (*p* = 0.06).

### 3.2. Multiple Regression Analysis

In a linear multivariable regression analysis, PA, when considered as a two-class variable (sedentary vs. active), was an independent predictor of EP (*p* = 0.001). After the inclusion of heart rate in the regression, PA remained a significant predictor of EP, but the association was weakened with a *p*-value = 0.04 (Table 2). Moreover, the association of PA with PWVβ (*p* < 0.001) was attenuated after adjusting for heart rate (*p* = 0.007, Table 3). In both regressions, heart rate was a strong predictor of carotid stiffness parameters (*p* < 0.001 for both EP and PWVβ) and improved the fit of the models (Table 4). The models, including heart rate, had the smallest AIC with a Delta AIC of 9.4 and 9.2, respectively, compared to the base model with covariates. Compared to the base model, both models, including heart rate, also had a smaller BIC, lending further support to the latter as being the most informative model.

## 4. Discussion

Our data confirm that aerobic PA has beneficial effects on carotid stiffness parameters and extend previous observations by showing that the negative association between the level of PA and carotid stiffness is linear in normotensive individuals and U-shaped in hypertensive patients. Indeed, within the hypertensive subgroup, the lowest stiffness parameters were observed in the participants performing leisure-time PA. 

An association between PA and large artery stiffness has been previously shown in cross-sectional [19,20,21] as well as intervention studies [22,23], but has never been evaluated in relation to the intensity of exercise training before. In keeping with our results, Sugawara et al. showed that the carotid stiffness parameter β significantly decreased after moderate or vigorous intensity cycling exercise training in a small number of apparently healthy sedentary or recreationally active women [21]. However, due to the small number of participants, no comparison was made between the two exercise modalities, and no data were available for the hypertensive subjects. A beneficial effect of a 24-week lifestyle intervention, including aerobic exercise and Mediterranean-style diet, on carotid stiffness parameter β was found by Aizawa et al. in 63 subjects with metabolic syndrome [24]. However, in that study, no information was available as to the effect of different exercise intensities on carotid stiffness. In addition, all intervention studies were conducted over a short period of time and thus may not be representative of the chronic effects of PA on carotid artery properties. In the present study, the participation of our active subjects in the respective PA category lasted for at least 2 years, making it likely that the association with carotid stiffness parameters was the result of chronic exposure to exercise.

The beneficial effects of regular PA on the cardiovascular system have been documented by strong experimental and epidemiological evidence [25,26]. A lifestyle of regular exercise and training has been shown to reduce the risk of cardiovascular events and mortality [27,28]. Several studies have found a graded association between PA and outcomes, showing no additional benefits after reaching a certain threshold [10,29], and some studies even showed a U-shaped association between PA and mortality [30]. In a study of 55,000 participants from the Aerobics Center Longitudinal Study, substantial benefits were obtained with PA doses much lower than those recommended by current major exercise guidelines [31]. In addition, it is still largely unknown whether PA dosage should differ for certain groups of individuals [32]. It is thus important to identify the level of PA that can provide the optimal effect on vascular structure and function in different settings of health and disease.

To this end, we chose two clear-cut groups of individuals trained in aerobic sports. Considering how anamnestic information on PA is often unreliable, to reduce the common biases observed in previous studies of PA [33], competitive athletes were enrolled because they train at a higher intensity and volume than individuals involved in recreational non-competitive sports. The results obtained in the normotensive athletes are reassuring because they indicate that participation in competitive athletics is beneficial in healthy individuals with a normal BP.

Hypertension is often associated with vascular dysfunction. An increased BP leads to vasoconstriction and alterations in the morphology of the small arteries and arterioles due to changes in vascular smooth muscle and endothelial cells, extracellular matrix, and perivascular tissues [34,35]. This leads to the impairment of vascular distensibility [34,35] with reduced flow reserve at the level of the coronary [36] and cerebral microcirculation [37]. Capillary rarefaction and remodeling are other morphological changes observed in hypertensive animals and human beings, which contribute to increased vascular resistance [38,39]. In this milieu, it is possible that a high level of chronic PA provides no benefit or even causes a detrimental effect on vascular structure and function. An exercise-induced exacerbation of oxidative stress has been shown to occur in hypertensive patients compared to healthy controls, and reactive oxygen species have an important role in the development and time-course of hypertension [40]. Previous studies have shown that aortic stiffness appears to be resistant to change in response to short-term aerobic exercise interventions among subjects with pre- or stage 1 hypertension, such as those investigated in the present study [41]. In a study by Kraft et al., aortic stiffness was significantly lower in the fit, normotensive participants versus the unfit ones [42]. By contrast, in the hypertensive cohort, stiffness was not significantly different between the fit and unfit subgroups. However, in this as well as other previous studies, no information was provided as to the effect of different levels of exercise intensity or physical fitness on large artery stiffness. Our results show that high-intensity training had virtually no effect on carotid stiffness parameters, which in the hypertensive athletes were similar to those in the sedentary subjects. In contrast, a beneficial effect was observed in the hypertensive participants performing recreational PA.

### Role of Heart Rate

In the present study, heart rate was a strong determinant of carotid artery stiffness, in agreement with the results of previous investigations. Experimental studies have shown that a progressive increase in heart rate with pacing in human beings was associated with a progressive increase in pulse wave velocity (PWV), indicating a cross-sectional association of heart rate with arterial stiffness [43,44]. According to Tan et al., there is a 0.17 m/s increase in PWV for each 10 beat/min increment in heart rate by pacing [44]. A direct association between heart rate and PWV was also found by Tomiyama et al. [45] and by Benetos et al. [46] in longitudinal studies both in normotensive and hypertensive subjects. It is thus likely that the decrease in heart rate caused by regular aerobic training is a main determinant of improved arterial distensibility in physically active subjects. Indeed, in the present study, the inclusion of heart rate in the regression models improved the model fit substantially and attenuated, but did not abolish, the relationship between PA and stiffness parameters. This suggests that low heart rate and PA independently exert beneficial effects on arterial elasticity and that PA-induced bradycardia only partially accounts for vascular function improvement. A regular PA may improve arterial elasticity through mechanisms other than heart rate reduction by protecting the arterial tree from the adverse effects of traditional risk factors for cardiovascular disease. The regression of microvascular remodeling, normalization of capillary density and the improvement of endothelial functions have been reported in hypertensive animals and humans after exercise training [47,48,49,50,51].

## 5. Limitations

A limitation of our study is that we used only two PA categories and could not provide a more graded relationship between the PA dose and its effects on carotid stiffness. Another major limitation is the cross-sectional nature of our study design that precludes us from making inferences on temporality. Third, we used brachial BP for the calibration of carotid diameter changes, which usually overestimates the central BP in an age-dependent manner. However, mean age was similar in the three PA groups.

## 6. Conclusions

There is still uncertainty as to the optimal exercise regimen required to achieve the greatest benefits in terms of cardiovascular health. The data of the present study indicate that in hypertension, there is a U-shaped relationship between the PA level and carotid stiffness, suggesting that leisure-time aerobic PA is more beneficial than high-intensity PA on arterial function. A high level of chronic PA provides no benefit to vascular structure and function in people with mildly elevated BP levels and might even cause a detrimental effect in patients with more severe hypertension [40,52]. The effect of exercise training on carotid stiffness seems to be partly, but not entirely, mediated by the reduction in heart rate that accompanies physical training. Future studies are required to validate our data in a larger series of subjects with a wider BP range and to explore the mechanisms underpinning these findings.

## Figures and Tables

**Table 1:** Characteristics of the 3 groups of study participants.

	Sedentary	Leisure-Time PA	Competitive Athletes	*p*-Value
N	120	120	120	
Age, years	33.9 (13.1)	31.8 (12.8)	29.6 (14.1)	0.042
Sex, men	77.5%	77.5%	83.3%	0.435
BMI, kg/m^2^	24.4 (3.7)	23.7 (3.7)	22.8 (2.8)	0.002
Office Systolic BP, mmHg	123.8 (12.5)	123.9 (12.8)	122.2 (12.4)	0.57
Office Diastolic BP, mmHg	75.5 (10.2)	73.8 (10.3)	72.2 (9.8)	0.19
Heart rate, bpm	70.6 (11.1)	66.1 (9.3)	59.5 (12.5)	<0.001
Smoking, yes	10.0%	17.5%	7.5%	0.043

Data are mean (SD). PA indicates physical activity; BP indicates blood pressure.

**Table 2:** Predictors of pressure-strain elastic modulus in a multivariable linear regression analysis. Data from 360 subjects.

Predictors	Coefficient	Standard Error	T	*p*-Value	R Partial
Age, years	1.1225	0.0886	12.664	<0.0001	0.5595
Sex	3.6221	2.7703	1.307	0.19	0.0695
Body mass index, Kg/m^2^	0.5522	0.3482	1.586	0.11	0.0842
Smoking, yes/no	3.4167	3.1723	1.077	0.28	0.0573
Systolic blood pressure, mmHg	0.5364	0.0896	5.986	<0.0001	0.3040
Physical activity, no/yes	−4.7026	2.3142	−2.032	0.042	−0.1077
Heart rate, bpm	0.3189	0.0947	3.367	0.0008	0.1766

**Table 3:** Predictors of one-point pulse wave velocity in a multivariable linear regression analysis. Data from 360 subjects.

Predictors	Coefficient	Standard Error	T	*p*-Value	R Partial
Age, years	0.0417	0.0029	14.337	<0.0001	0.6072
Sex	0.1700	0.0909	1.870	0.062	0.0992
Body mass index, Kg/m^2^	0.0260	0.0114	2.273	0.024	0.1203
Smoking, yes/no	0.1050	0.1041	1.008	0.31	0.0537
Systolic blood pressure, mmHg	0.0184	0.0029	6.251	<0.0001	0.3161
Physical activity, no/yes	−0.2054	0.0760	−2.703	0.007	−0.1426
Heart rate, bpm	0.0104	0.0031	3.338	0.0009	0.1752

**Table 4:** Akaike information criterion (AIC), Delta AIC, and Bayesian information criterion (BIC) for the linear regression analyses having EP and PWVβ as dependent variables.

Models	AIC	Delta AIC	BIC
EP			
Model including PA	3386.7		3398.4
Base Model * (M0)	3163.9	222.8	3195.0
Model 1 (M0 + Heart rate)	3154.5	9.4	3189.5
PWVβ			
Model including PA	968.3		978.0
Base Model * (M0)	703.9	264.4	735.0
Model 1 (M0 + Heart rate)	694.7	9.2	729.6

* Including: PA, Age, Gender, Body mass index, Systolic blood pressure, and Smoking. AIC indicates Akaike Information Criterion; Delta AIC is a measure of each model relative to the model with the smallest AIC; BIC indicates Bayesian Information Criterion. EP indicates pressure-strain elastic modulus. PWVβ indicates one-point pulse wave velocity. PA indicates physical activity level (two-class category).

## Data Availability

The data that support the findings of this study are available on request from the corresponding author.
